# Supplementary figures and images for: Cold temperature induces a TRPM8-independent calcium release from the endoplasmic reticulum in human platelets
Source: PLoS One. 2024 Mar 4;19(3):e0289395. doi: 10.1371/journal.pone.0289395 (PMC10911599; doi:10.1371/journal.pone.0289395)

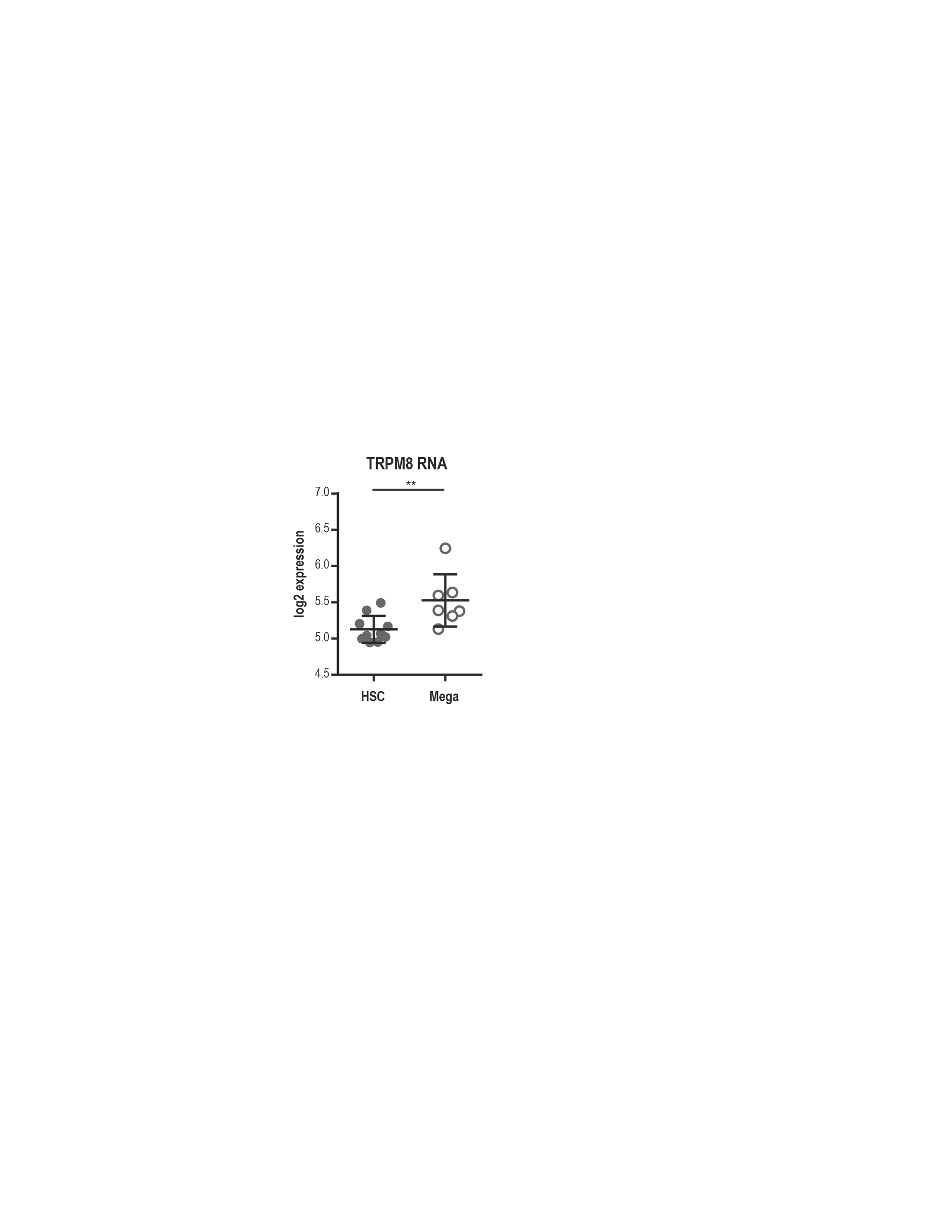

Supplement: S1 Fig — Data were obtained from BloodSpot, a gene-centric database of mRNA expression of hematopoietic cells (Bagger et al., 2018). RNAseq was performed by Novershtern et al., 2011, source: GSE24759. The HSCs were identified as CD133-positive and CD34-dim (n = 10), while Megakaryocytes as CD34+, CD41+, CD61+, and CD45-negative (n = 7). Error bars indicate Mean ± SEM. Statistical analysis was performed using an unpaired Student t-test, where asterisks indicated a p = 0.009. (TIF) [file pone.0289395.s001.tif]

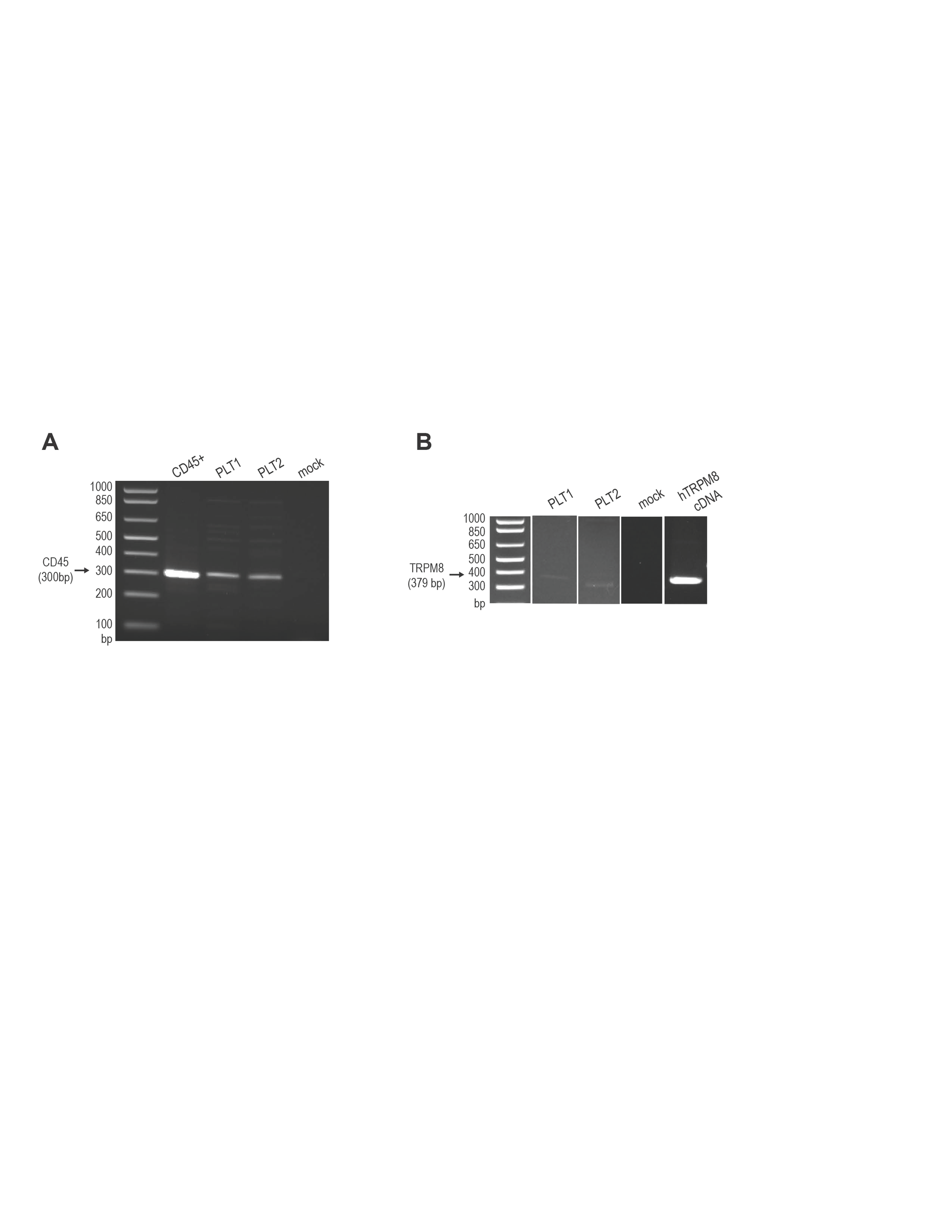

Supplement: S2 Fig — Agarose gel electrophoresis of PCR products from CD45-and CD235a (Glycophorin A)-depleted platelet preparation. PRP was depleted of CD45-positive cells using magnetic microbeads using AutoMACS sorter. A. PCR reaction using CD45 primers from CD45-positive cells, platelet preparations from two separate donors, and mock. B. PCR reaction using the TRPM8 1410F/1788R primer set. Arrows indicate the size of the expected amplicons: 300bp for the CD45 primer set; and 379 bp for the 1410F/1788R primer set. (TIF) [file pone.0289395.s002.tif]

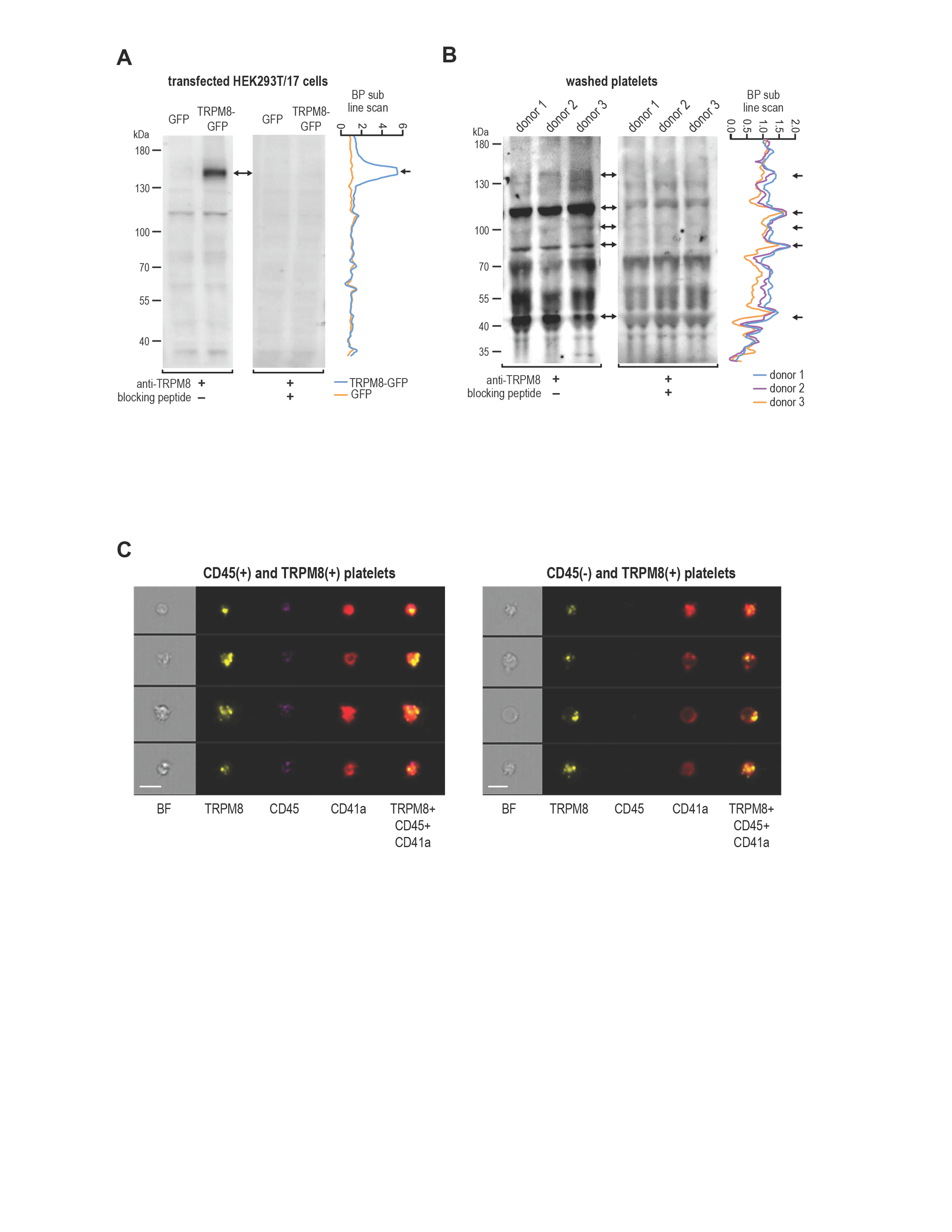

Supplement: S3 Fig — A. Western blot from HEK293T/17 cell lysates, transfected with GFP or TRPM8-GFP. Anti-TRPM8 (ACC-049) was used with or without blocking peptide (BLP-CC049). The expected size for TRPM8-GFP fusion protein is ~160kDa (two ways arrow). Blocking peptide subtracted line scan (BP sub line scan) was calculated by measuring pixel intensity along a line drawn down the lanes, normalizing to a background at high molecular weight, and subtracting the values measured for the corresponding lanes with blocking peptide (blue for TRPM8-GFP lane; orange for GFP). Arrow in the line scan indicates a full-length TRPM8-GFP protein. B. Western blot of washed platelet lysates from three healthy donors. Line scan was calculated as in A. Arrows indicate potential TRPM8 protein. C. Representative images of random TRPM8-positive platelets population and CD45 (-/+) staining from one healthy donor by imaging flow cytometry. 20,000 events were measured for each sample. The scale bar is 7 μm. (TIF) [file pone.0289395.s003.tif]

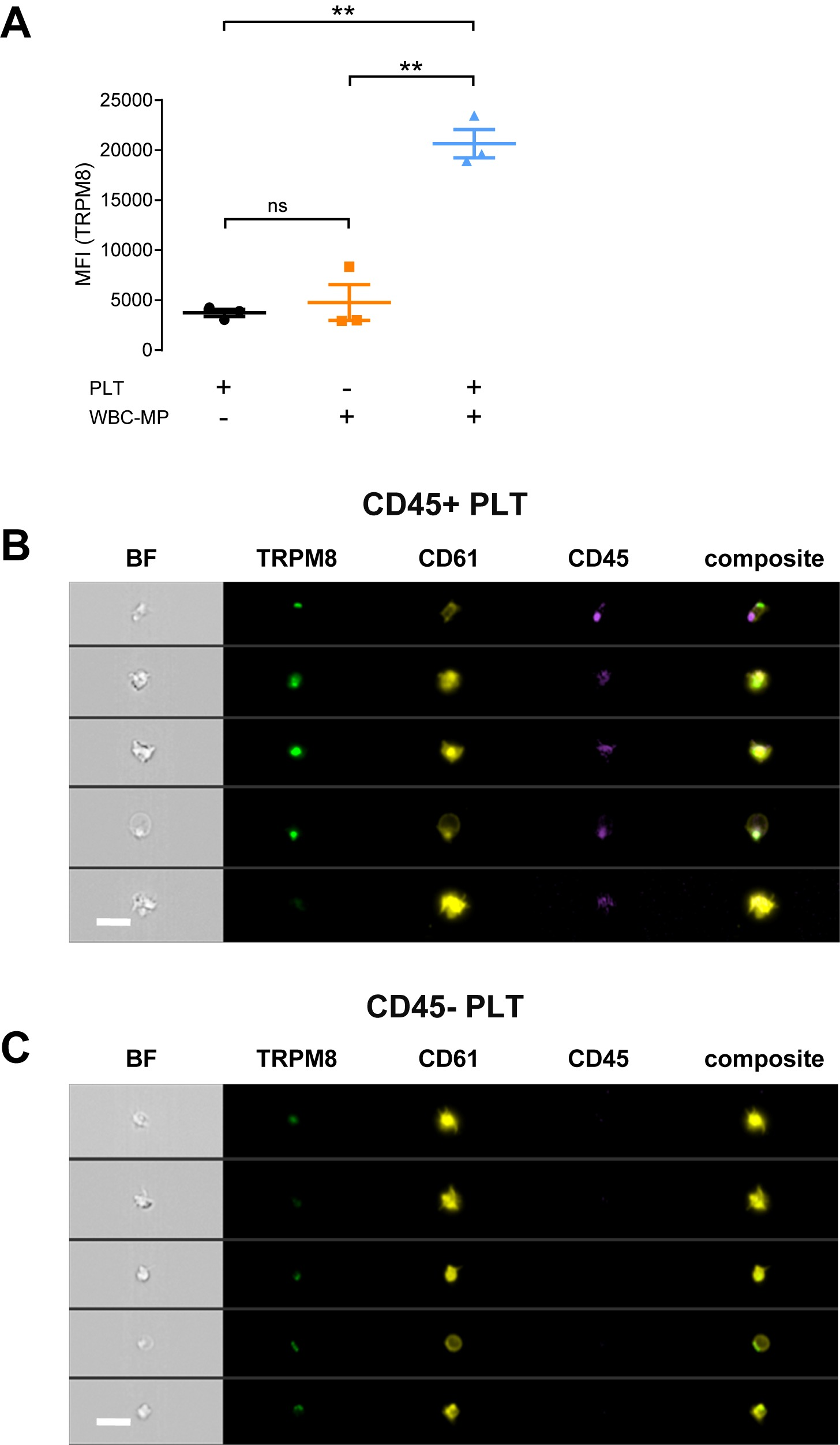

Supplement: S4 Fig — (A-C) TRPM8 expression in platelets, WBC-derived microparticles, and platelets incubated with WBC-derived microparticles. Platelet-rich plasma was incubated with WBC-derived microparticles for 15 min at RT. Platelet-rich plasma alone, WBC-derived microparticles alone, and platelet-rich plasma incubated with WBC-derived microparticles were stained with TRPM8 antibody with a secondary FITC-labeled antibody, and the pan WBC CD45 antibody, along with CD61 and read by conventional flow cytometry (A) and imaging flow cytometry (B-C). B. Representative CD45 positive platelets (CD61+ and FSC/SSC gated). C. Representative CD45 negative platelets (CD61+ and FSC/SSC gated). Data in (A) are shown as individual data points, mean ± standard error of the mean. N = 3 independent experiments.**p = 0.0029 for PLT versus PLT+WBC-MP, and **p = 0.0037 for WBC-MP versus PLT+WBC-MP. Ns = not significant (p = 0.88). Statistical analysis was performed using an One Way ANOVA with Tukey correction for multiple comparisons and assumed equal sphericity. White bar indicates 7μm. (TIF) [file pone.0289395.s004.tif]

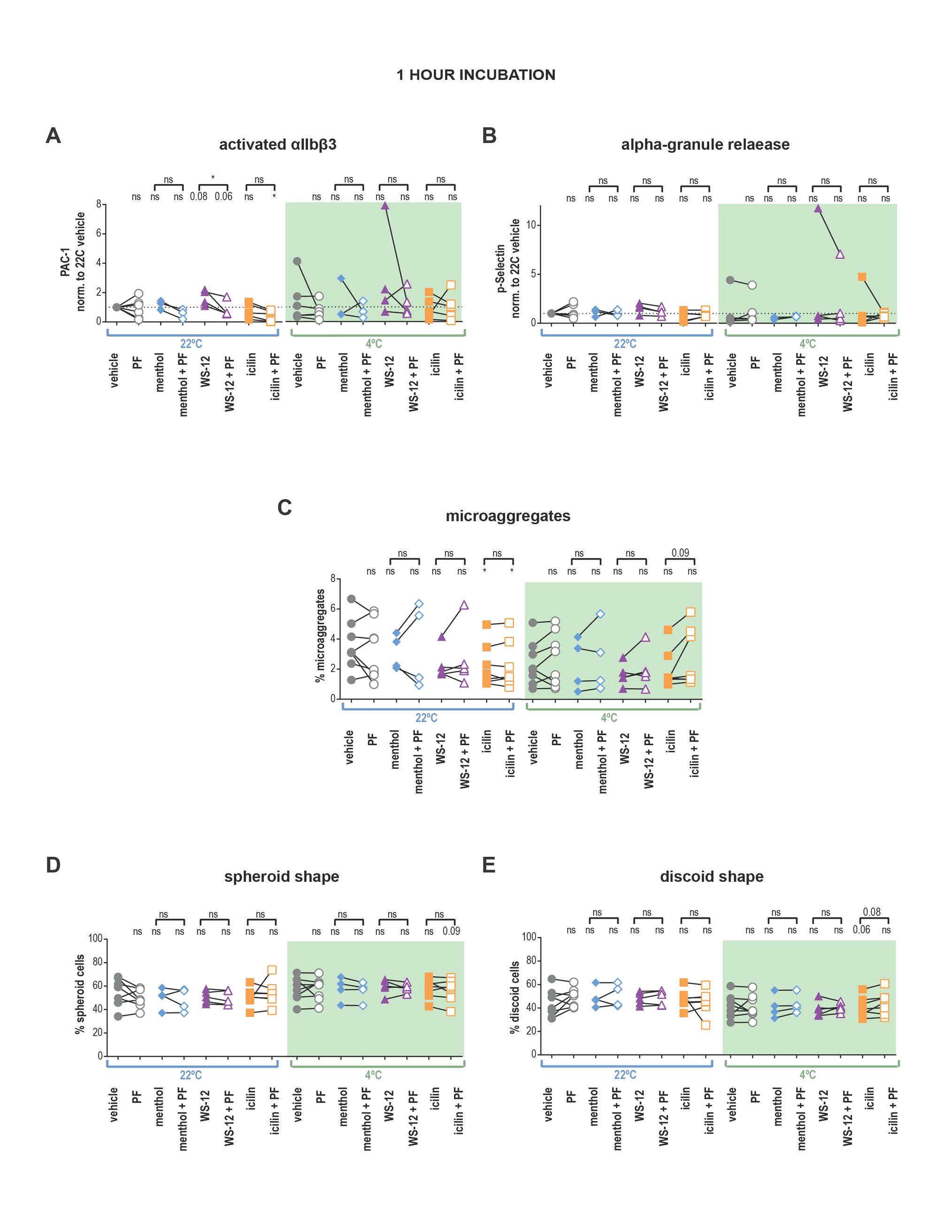

Supplement: S5 Fig — (A-B) Change in Calcium Green™-1 fluorescence levels baseline subtracted and normalized to maximum obtained after addition of calcium ionophore 7 μM A23187. HEK293T/17 cells transfected with TRPM8 (A) or empty vector (B), untransfected) were suspended in HEPES buffered saline containing either 0 mM Ca2+ and 100 μM EGTA (black) with vehicle DMSO, 2 mM Ca2+ with vehicle DMSO (gray) or 2 mM Ca2+ with 2 μM PF 05105679 (blue). C. Quantification of maximal calcium increase at 10˚C in HEK293T/17 cells, n = 2. D. The overlay of the linear fit (dashed line, R2 = 0.82) of the average negative control—the un-transfected HEK cells in 2 mM Ca2+ (same as in B, gray) and the average calcium response in washed platelets in 0 mM Ca2+ and 100 μM EGTA -containing Tyrode’s buffer with vehicle DMSO (gray, same as Fig 7C) or 5 μM thapsigargin (pink, same as Fig 7C). Arrow indicates an apparent threshold for platelet activation at ~ 23˚C. (TIF) [file pone.0289395.s005.tif]

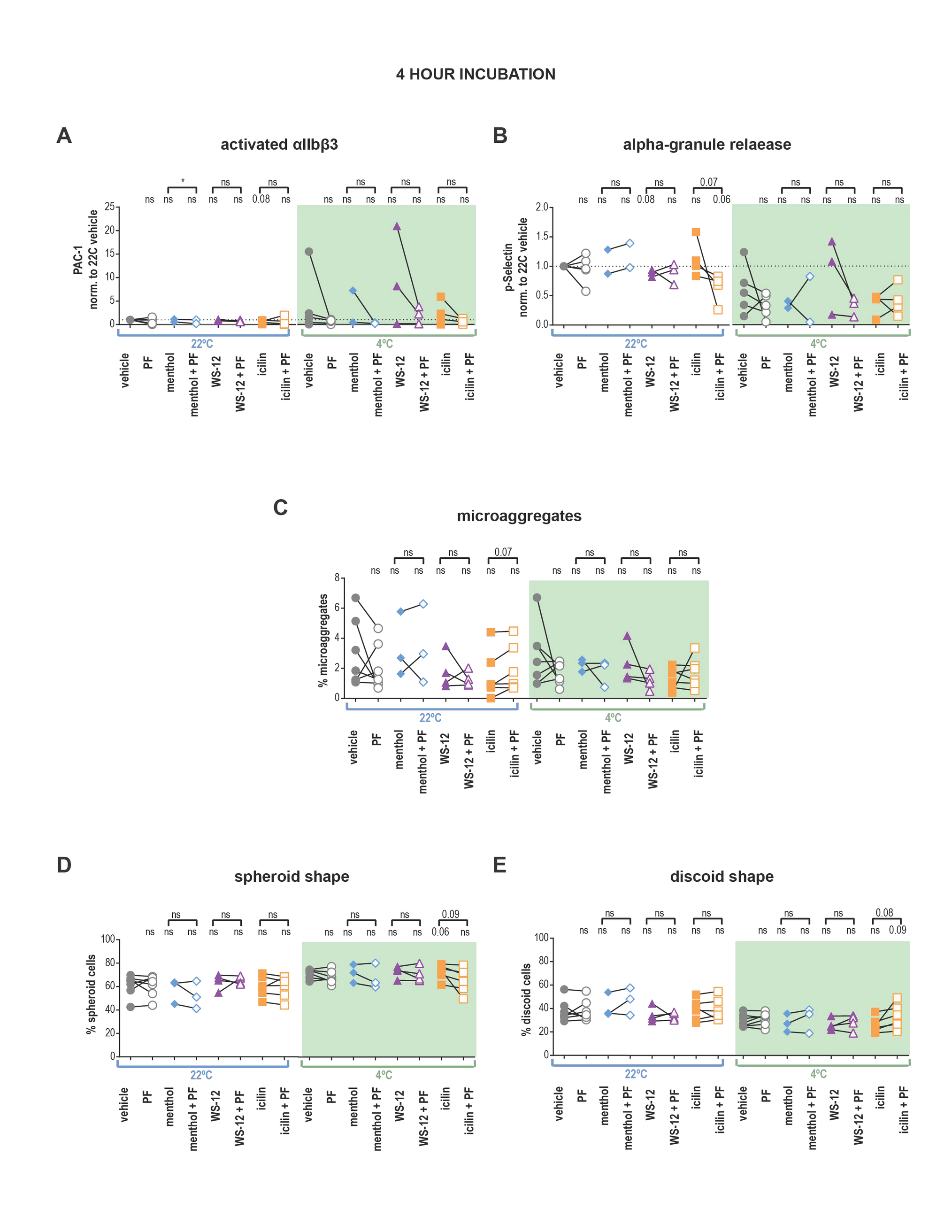

Supplement: S6 Fig — (A-B) Human washed platelets were evaluated via flow cytometry. A. Integrin αIIbβ3 activation in samples stained with PAC-1 fluorescent antibody (MFI normalized to vehicle). B. Alpha granule release as seen from P-selectin externalization (anti-P-selectin fluorescent antibody MFI normalized to vehicle). Initially, samples were pre-incubated with either vehicle DMSO or PF 05105679 (2 μM) for 5 minutes. Next, samples were treated with either vehicle (Ethanol), menthol (500 μM), WS-12 (2 μM) or icilin (100 μM) for 1 hour at either 22˚C (white background) or 4˚C temperature (green background). Values were normalized to those measured in platelets treated with the vehicle at 22˚C. (C-E) Samples were evaluated via imaging flow cytometry. C. Percent microaggregates in samples treated the same as in A and B. (D, E) Percent spheroid (D) or discoid (E) cells in samples treated same as in A and B. Lines connecting data points indicate the same donor. Statistical analysis was performed using paired Student t-test, where asterisks indicated a p-value lower than 0.05 for *, and “ns” indicates s p-value >0.05. Symbols above brackets indicate paired comparison between treatment groups, and without bars indicate comparison to vehicle. (TIF) [file pone.0289395.s006.tif]

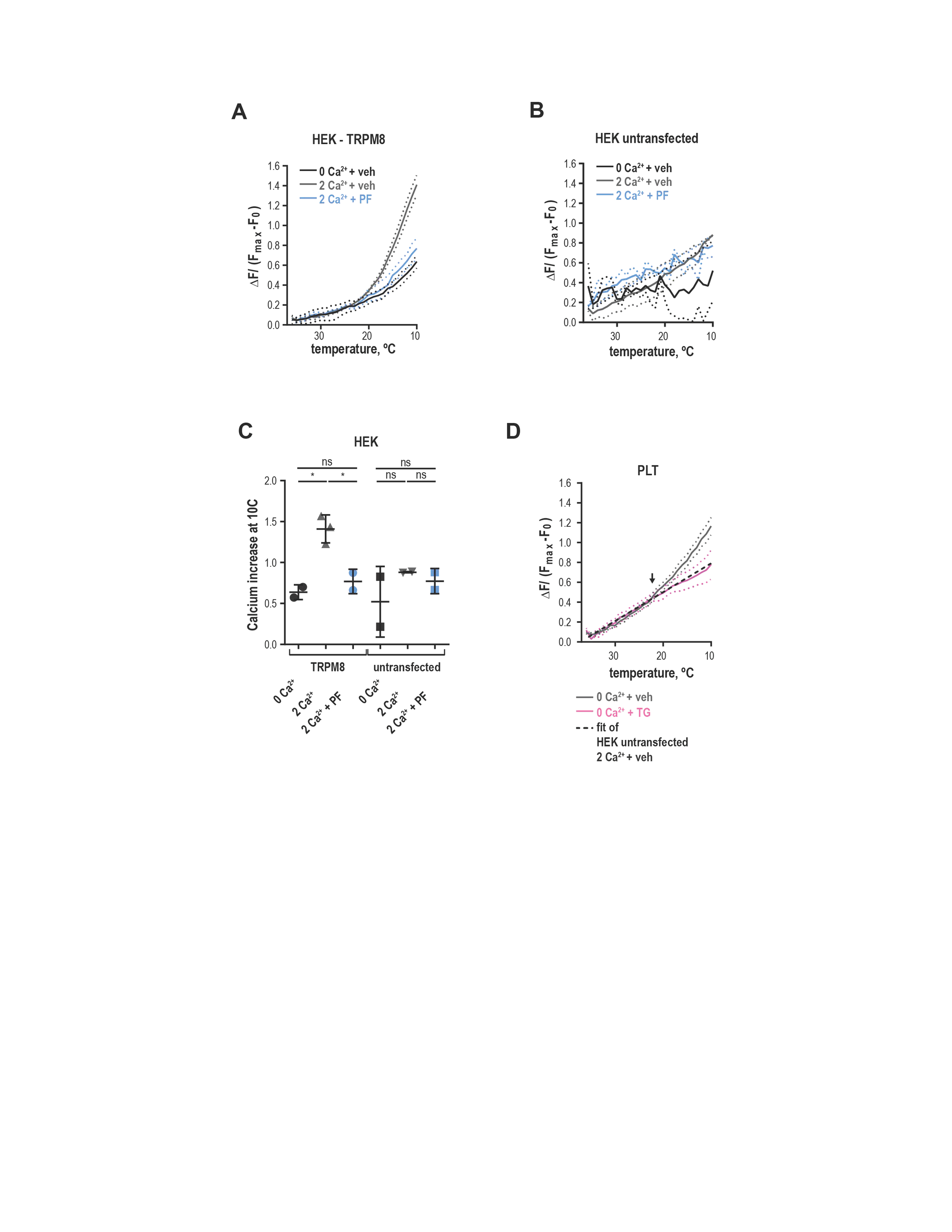

Supplement: S7 Fig — (A-B) Human washed platelets were evaluated via flow cytometry. A. Integrin αIIbβ3 activation in samples stained with PAC-1 fluorescent antibody (MFI normalized to vehicle). B. Alpha granule release as seen from P-selectin externalization (anti-P-selectin fluorescent antibody MFI normalized to vehicle). Initially, samples were pre-incubated with either vehicle DMSO or PF 05105679 (2 μM) for 5 minutes. Next, samples were treated with either vehicle (Ethanol), menthol (500 μM), WS-12 (2 μM) or icilin (100 μM) for 4 hours at either 22˚C (white background) or 4˚C temperature (green background). Values were normalized to those measured in platelets treated with vehicle. (C-E) Samples were evaluated via imaging flow cytometry. C. Percent microaggregates in samples treated the same as in A and B. (D, E) Percent spheroid (D) or discoid (E) cells in samples treated the same as in A and B. Lines connecting data points indicate the same donor. Statistical analysis was performed using paired Student t-test, where asterisks indicated a p-value lower than 0.05 for *, and “ns” indicates s p-value >0.05. Symbols above brackets indicate paired comparison between treatment groups, and without bars indicate comparison to vehicle. (TIF) [file pone.0289395.s007.tif]

Fig.1.A

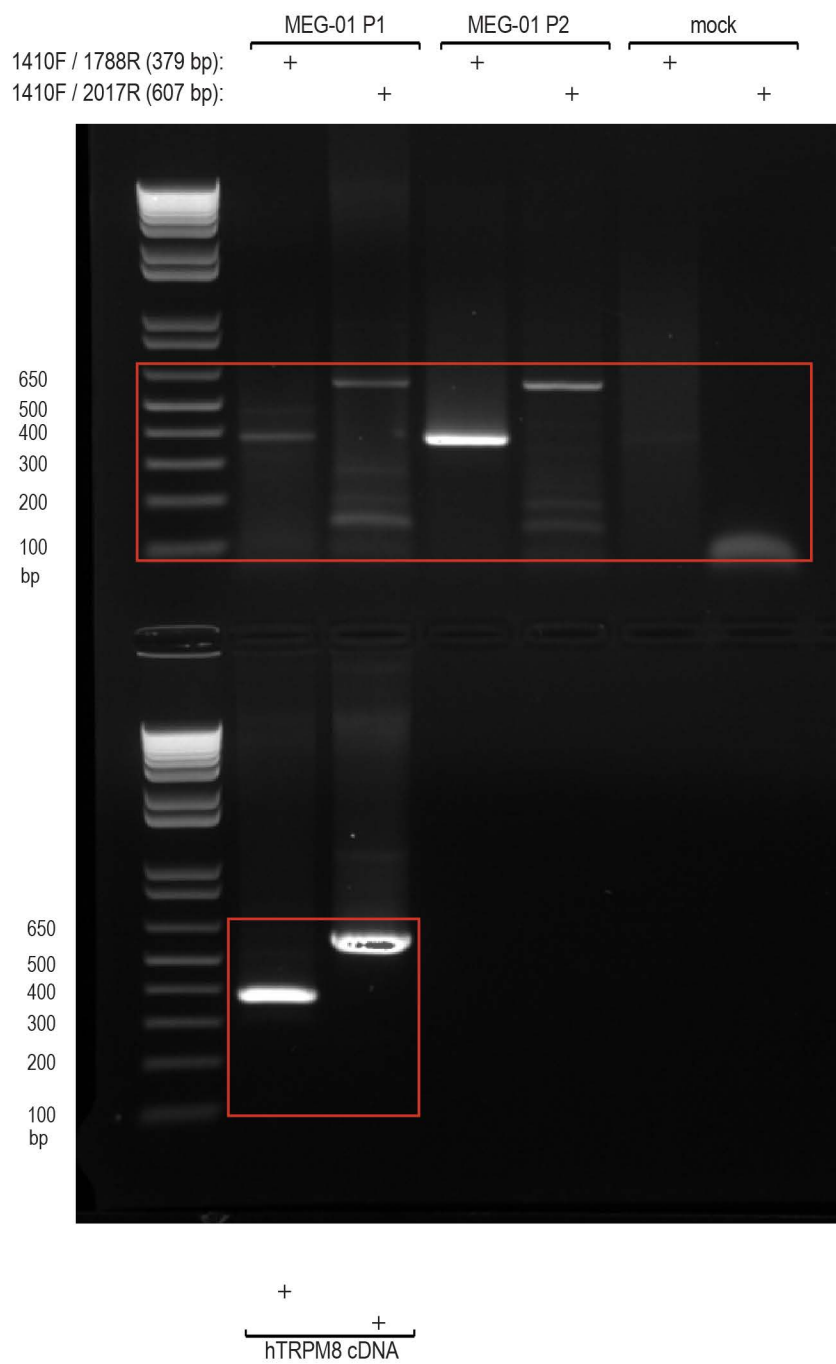

**Fig.1.B**

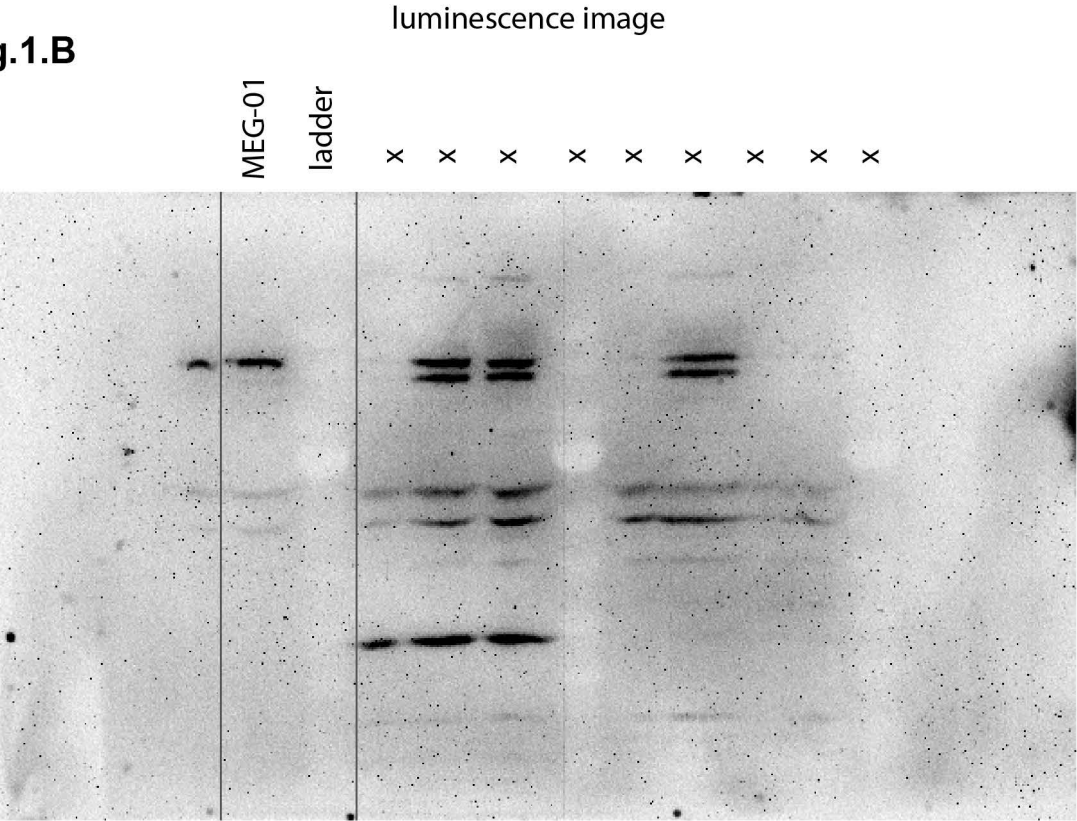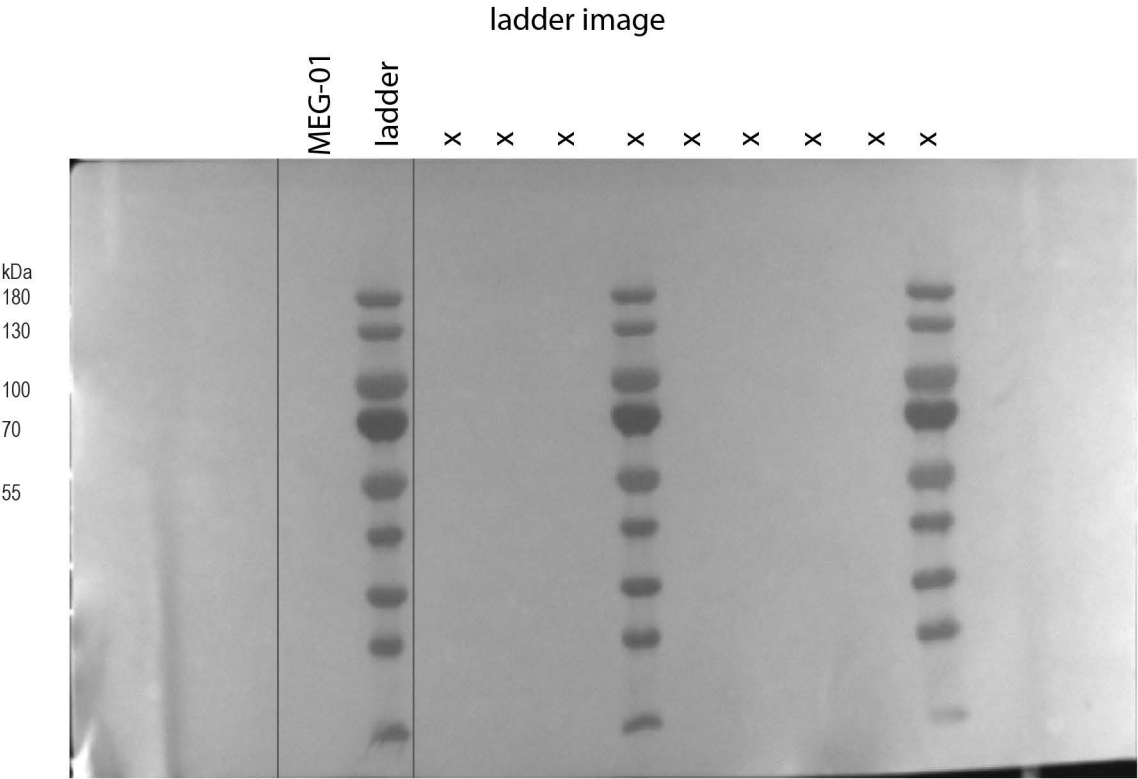

Suppl.Fig.2.A

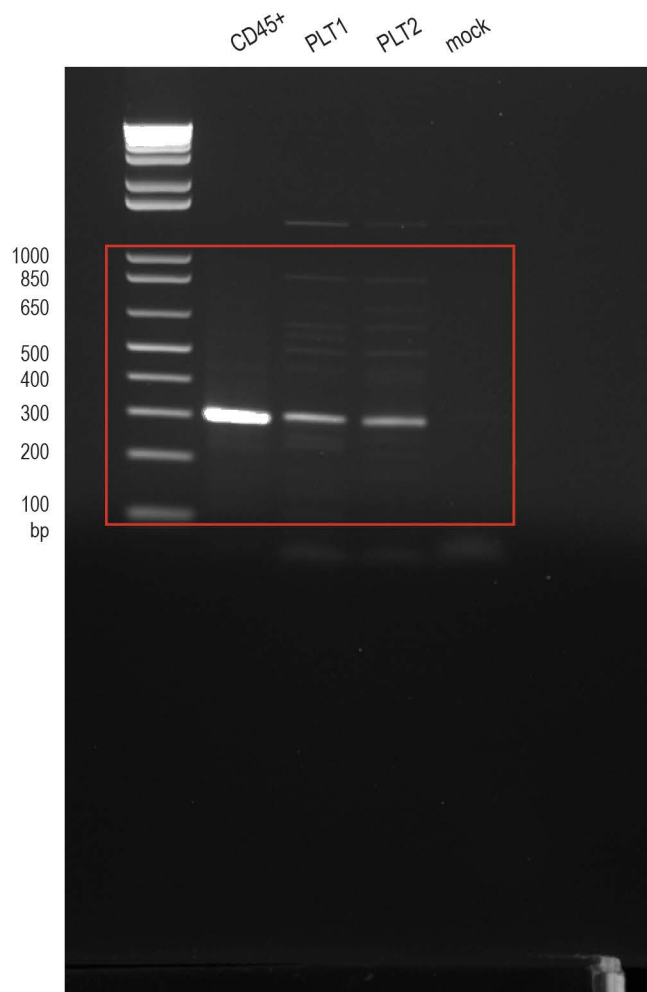

Suppl.Fig.2.B

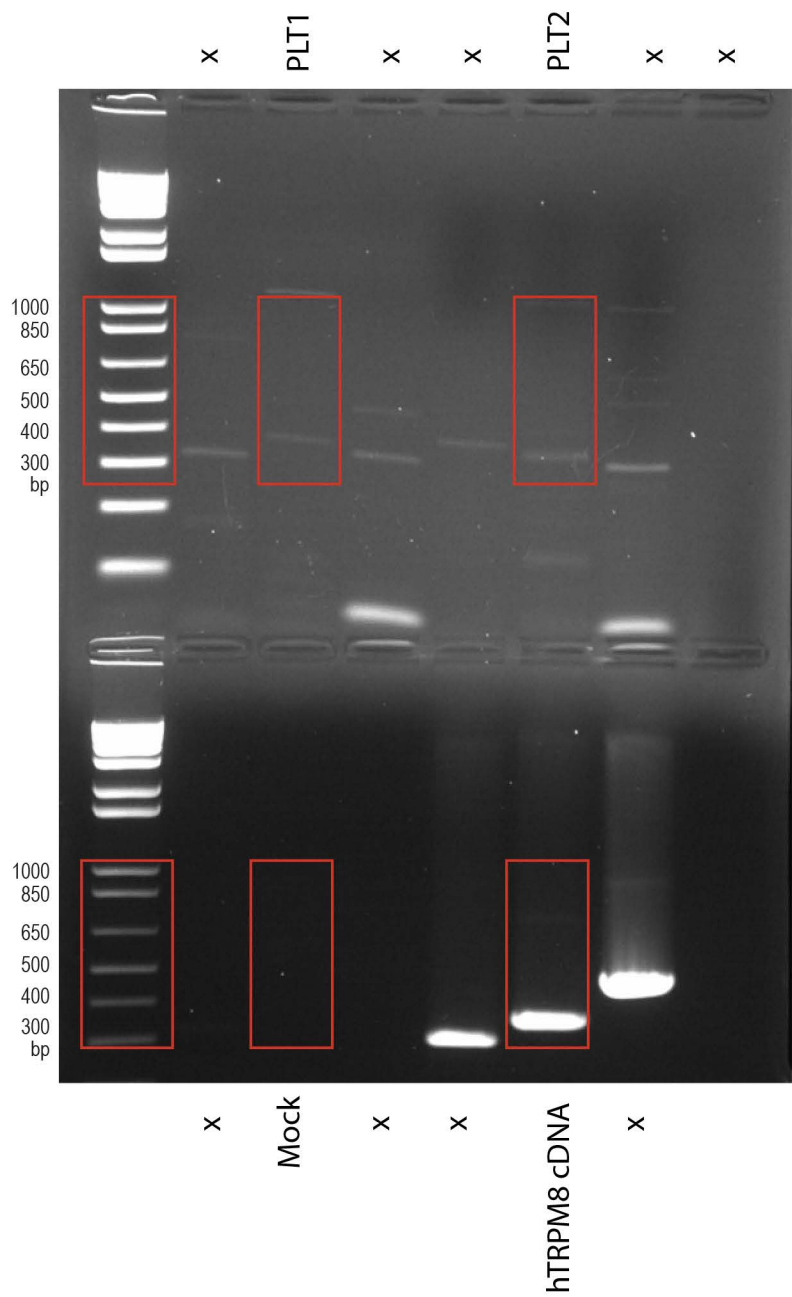

Suppl.Fig.3.A

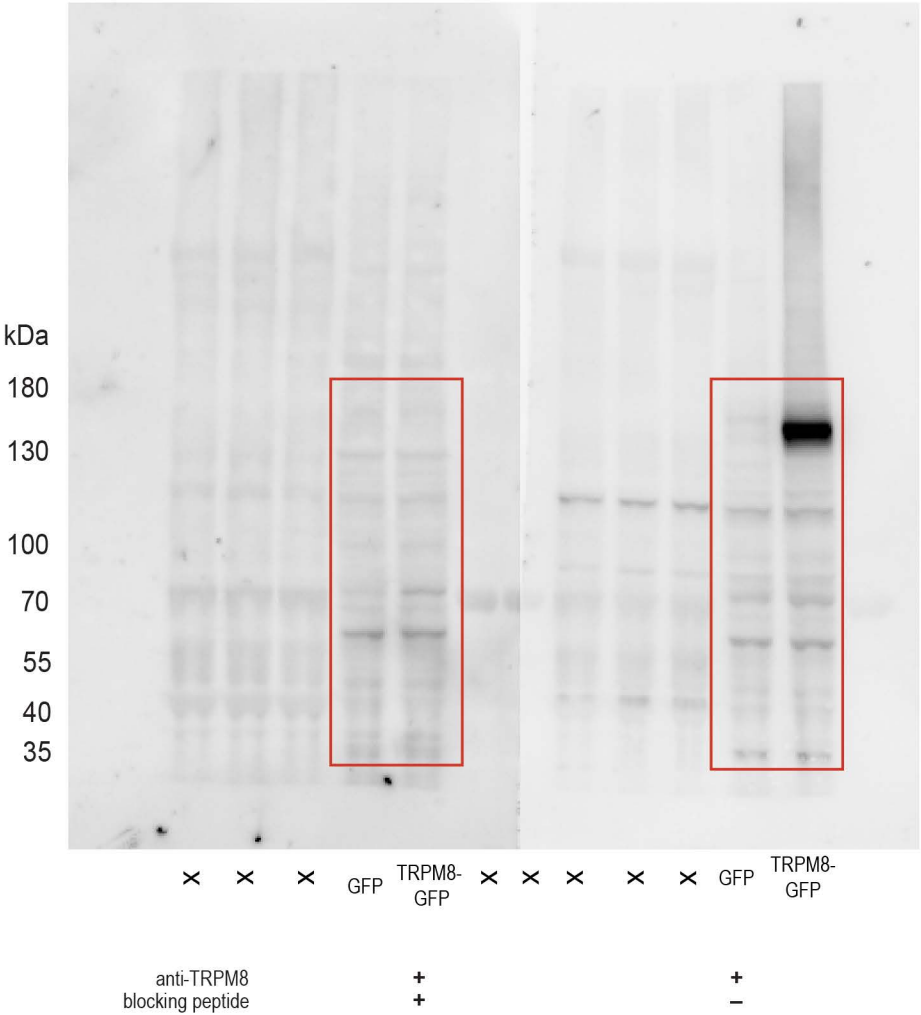

Suppl.Fig.3.B

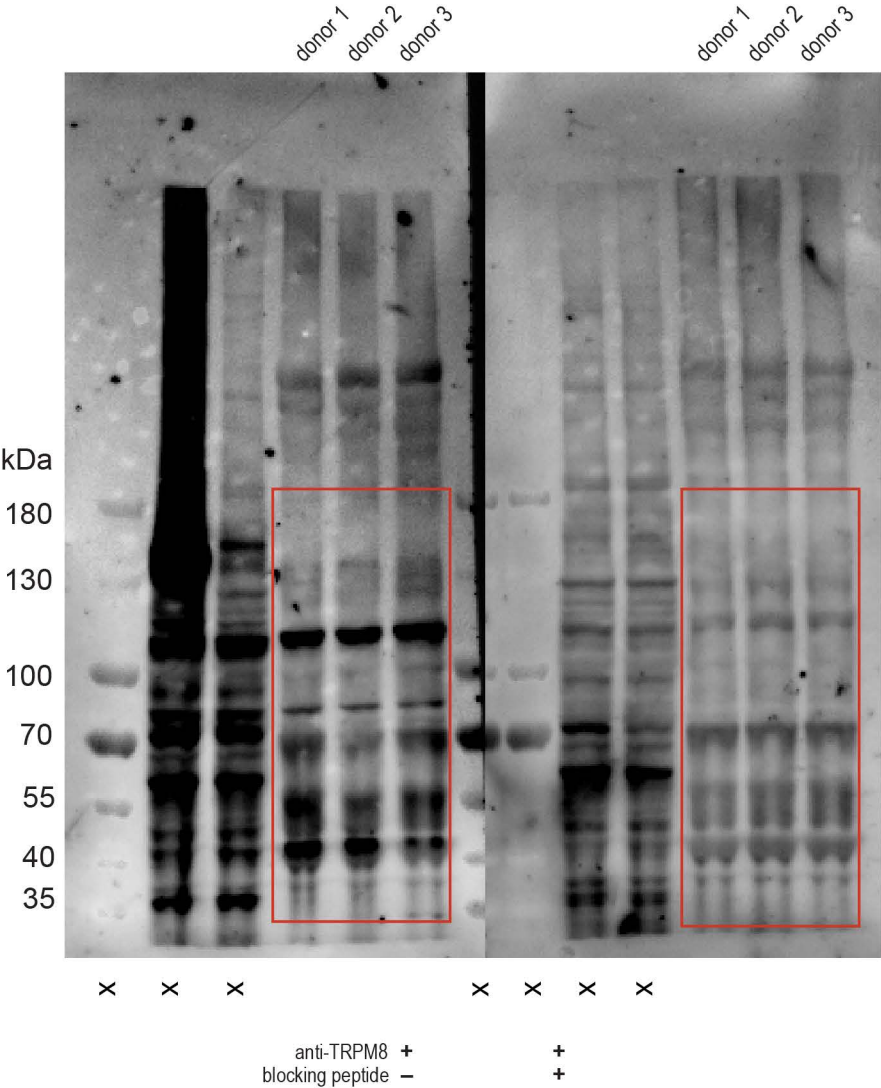

Supplement: S1 Raw images — (PDF) [file pone.0289395.s010.pdf]
