# Supplementary material for: Cold temperature induces a TRPM8-independent calcium release from the endoplasmic reticulum in human platelets
Source: PLoS One. 2024 Mar 4;19(3):e0289395. doi: 10.1371/journal.pone.0289395 (PMC10911599; doi:10.1371/journal.pone.0289395)
Supplement: S1 File — (DOCX) [file pone.0289395.s008.docx]

|  |  |  |  |
| --- | --- | --- | --- |

# Supporting Information

## TRPM8 mRNA in human platelets.

We examined purified human platelets for the presence of TRPM8 messenger RNA. We were able to amplify the TRPM8 message from platelets using PCR. However, despite electromagnetic bead depletion, we failed to rule out contaminating leukocytes as a possible source. It has been shown that some leukocyte populations are TRPM8-positive (Acharya et al., 2021). Indeed, in addition to TRPM8, we observed the leukocyte-specific CD45 expression from our supposedly leukocyte-depleted platelet preparations. This finding renders the source of the TRPM8 message questionable (Supplementary Fig 2).

## TRPM8 protein in human platelets

For the detection of TRPM8 protein expression in human platelets, we used an antibody specifically recognizing an extracellular epitope of TRPM8 (Supplementary Fig 3 A). We utilized a blocking peptide specific to this antibody, to aid in identifying the epitope-specific bands. We first tested the effectiveness of the blocking peptide and specificity of the antibody by comparing the lysates of HEK293T/17 cells expressing either TRPM8-GFP fusion protein (estimated at 160kDa, Supplementary Fig 3 A) or GFP control. Although several bands were present, the TRPM8-specific band was only present in TRPM8-GFP expressing cells and disappeared when the blocking peptide was included. We used a line scan down each lane to quantify the blots. We subtracted the line scans of the blots treated with blocking peptide from the corresponding anti-TRPM8 antibody-only treated blots (see Methods; Supplementary Fig 3 A, right). There was a single peak in the TRPM8-GFP line scan (blue) at the expected size for 160kDa (Supplementary Fig 3 A, arrow), which was not present in the GFP-only control (orange). Next, we probed for TRPM8 protein in platelet lysates of three different healthy donors (Supplementary Fig 3 B). We identified five bands that were potentially specific to the TRPM8 epitope: ~140, ~110, ~80, and ~45 kDa (Supplementary Fig 3 B, indicated by arrows). The faint band at ~140 kDa, clearly seen in donor 2, is consistent with the size range for full-length TRPM8 protein [[1](#biblioRef00),[2](#biblioRef01)]. Interestingly, we observed two relatively more intense bands ~80kDa and ~45kDa, which may correspond to less well-characterized shorter isoforms of TRPM8 [[3](#biblioRef02)]. In particular, the ~45kDa isoform was previously reported to be functional in the endoplasmic reticulum of prostate primary epithelial cells and keratinocytes [[4](#biblioRef03),[5](#biblioRef04)].

Because the TRPM8-specific bands were low intensity, we cannot rule out contamination from TRPM8-positive leukocytes. To address this issue, we stained platelets against the CD45 leukocyte marker and TRPM8 and subjected them to imaging flow cytometry. CD45-positive platelets comprised ~2% of the TRPM8-positive platelet population. We observed that TRPM8 punctate staining did not overlap with CD45 (Supplementary Fig 3 C). This indicates that TRPM8 punctate staining does not only have leukocyte origin.

## Leukocyte-derived microparticles as the source of TRPM8 in human platelets

We generated leukocyte-derived microparticles as previously described.[[6](#biblioRef05)] In brief, we obtained leukocytes by centrifuging heparinized whole blood at 1800g and isolating the buffy coat. Buffy coat leukocytes were stimulated with 10 µM A23187 and subsequently centrifuged at 1500g to isolate microparticles. The supernatant was decanted and incubated with washed platelets (generated as described above) for 15min. Anti-TRPM8 (Rabbit-anti human, Alomone labs #ACC-049) and secondary goat anti rabbit IgG FITC (Invitrogen#65-6111) were incubated along with a pan anti-platelet antibody (CD61-PE), and a pan anti-leukocyte antibody (CD45-V450) (both BD, Pharmingen, San Diego, CA, USA) for 15 min each, respectively and the sample was stopped by excess buffer and read immediately on a conventional and imaging flow cytometer.

## Platelets cannot be activated by TRPM8 agonists and cooling for 1 or 4 hours

Next, we tested whether prolonged incubation with TRPM8 agonists for 1 or 4 hours can activate platelets (Supplementary Fig 4, 5 A-B, white background). Compared to the vehicle, TRPM8 agonists did not significantly increase αIIbβ3 activation or α-granules (Supplementary Fig 4, 5 A-B). Although, for WS-12 there was an apparent trend for an increase in αIIbβ3 activation at 1 hour 22°C incubation (Supplementary Fig 4 A, p =0.08), which was significantly decreased by pre-incubation with TRPM8 inhibitor PF 05105679 (Supplementary Fig 4 A, p =0.04). Agonist-dependent TRPM8 channel opening is enhanced by lower temperatures (Zakharian et al., 2010). Therefore, we incubated platelets with TRPM8 agonists for either 1 or 4 hours at 4°C (Supplementary Figs 4 and 5, green background). We observed no significant increases in αIIbβ3 activation or degranulation of α-granules at 4°C (Supplementary Fig 4, 5 A-B, green background). TRPM8 agonists also did not change the microaggregate content (Supplementary Fig 4, 5 C) or shape distribution (Supplementary Fig 4, 5 D-E) in platelet samples, regardless of incubation time or temperature. Furthermore, pre-incubation with TRPM8 inhibitor PF 05105679 in samples with TRPM8 agonists did not affect the microaggregate content (Supplementary Fig 4, 5 C), or shape change (Supplementary Fig 4, 5 D-E). Taken together, we show that platelets cannot be activated by TRPM8 agonists.

## Cold-induced calcium response of TRPM8-expressing HEK293T/17 cells

To establish the protocol, we used a cell line HEK293T/17 transfected with either TRPM8 or empty vector cDNA. Supplementary Fig 6 A shows the average fluorescence increase with decreased temperature from HEK293T/17 cells transfected with TRPM8 cDNA. We observed a steep increase in response to chilling when 2 mM Ca2+ was included (Supplementary Fig 6 A), with a kink at approximately 23°C, consistent with the TRPM8 threshold for temperature activation (Fujita et al., 2013; Peier et al., 2002). In contrast, in the un-transfected control cells the temperature response curve was linear (Supplementary Fig 6 B, D). This temperature-dependent calcium increase in TRPM8 transfected cells was abolished when no extracellular calcium was present (Supplementary Fig 6 A, C). Furthermore, the addition of PF 05105679 in the presence of extracellular calcium diminished the temperature response in TRPM8 transfected (Supplementary Fig 6 A, C). Thus, we were able to record temperature-induced TRPM8-dependent calcium influx in transfected HEK293T/17 cells using real-time PCR.


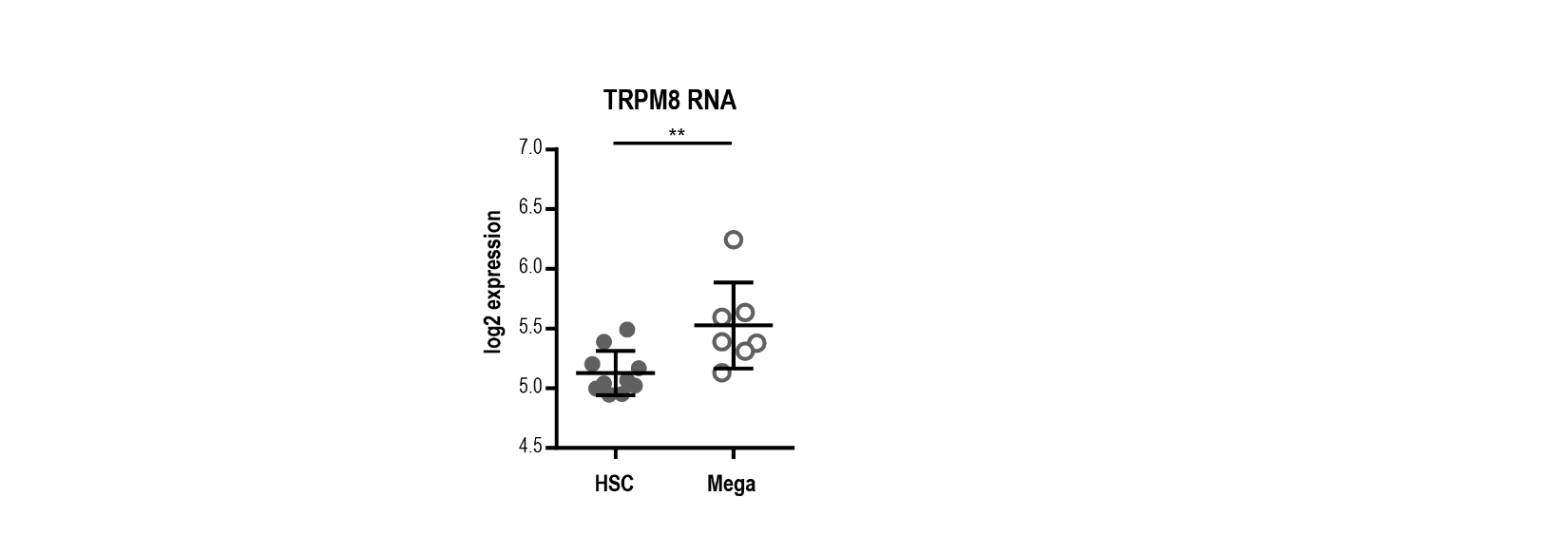


***Supplementary Fig 1. TRPM8 gene expression in megakaryocytic lineage during normal hematopoiesis.*** *Data were obtained from BloodSpot, a gene-centric database of mRNA expression of hematopoietic cells (Bagger et al., 2018). RNAseq was performed by Novershtern et al., 2011, source: GSE24759. The HSCs were identified as CD133-positive and CD34-dim (n=10), while Megakaryocytes as CD34+, CD41+, CD61+, and CD45-negative (n=7). Error bars indicate Mean ± SEM. Statistical analysis was performed using an unpaired Student t-test, where asterisks indicated a p = 0.009*


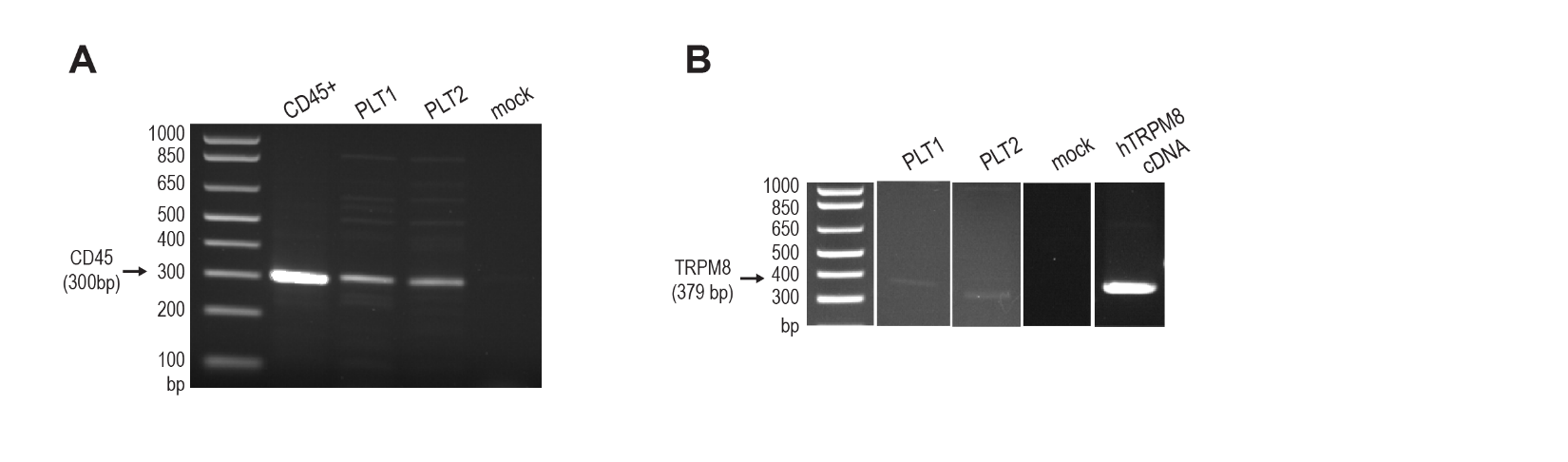


***Supplementary Fig 2. RBC- and WBC-depleted platelet preparation shows CD45 and TRPM8 signals.*** *Agarose gel electrophoresis of PCR products from CD45-and CD235a (Glycophorin A)-depleted platelet preparation. PRP was depleted of CD45-positive cells using magnetic microbeads using AutoMACS sorter.* ***A****. PCR reaction using CD45 primers from CD45-positive cells, platelet preparations from two separate donors, and mock.* ***B****. PCR reaction using the TRPM8 1410F/1788R primer set. Arrows indicate the size of the expected amplicons: 300bp for the CD45 primer set; and 379 bp for the 1410F/1788R primer set.*


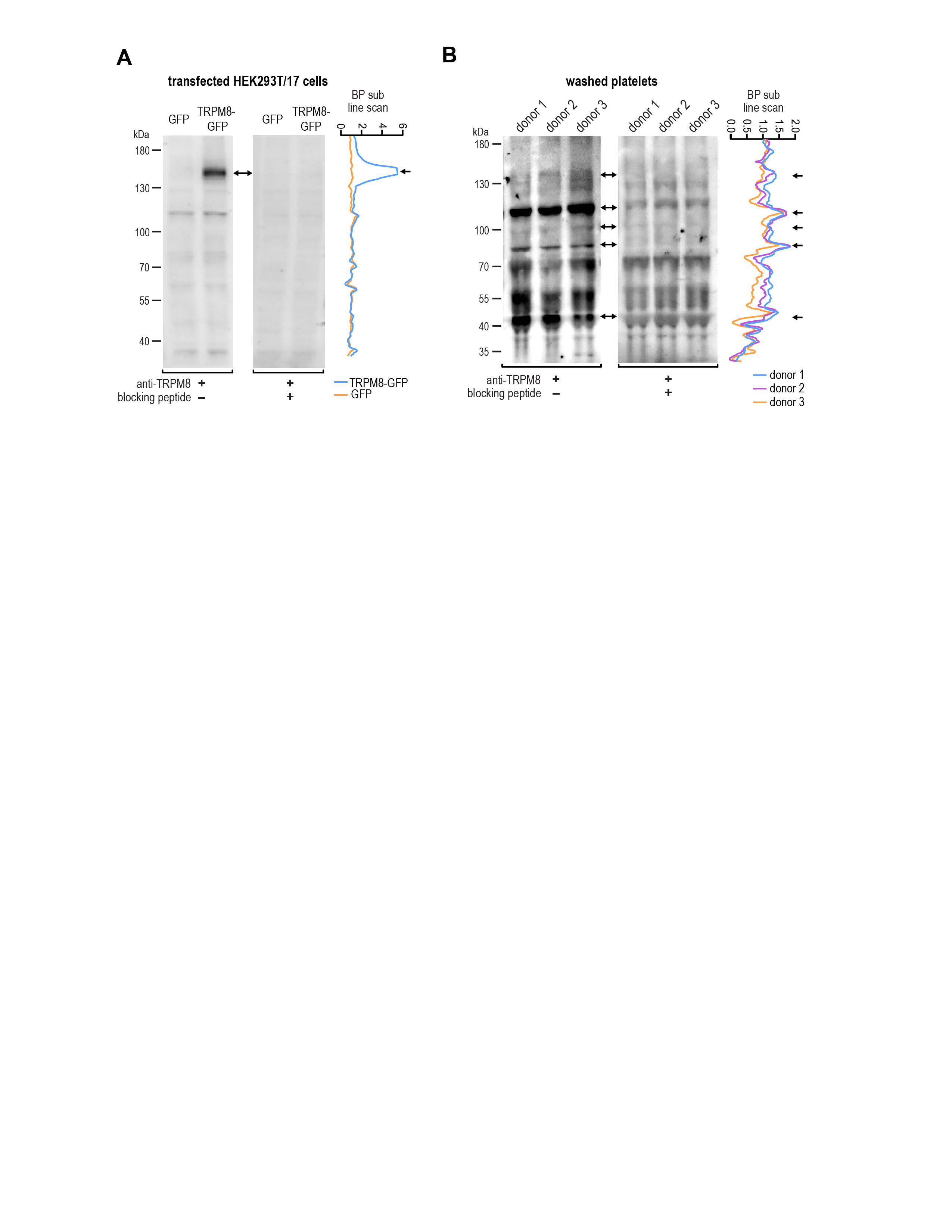


***Supplementary Fig 3. TRPM8 receptor protein in human platelets by immunoblotting.*** ***A****. Western blot from HEK293T/17 cell lysates, transfected with GFP or TRPM8-GFP. Anti-TRPM8 (ACC-049) was used with or without blocking peptide (BLP-CC049). The expected size for TRPM8-GFP fusion protein is ~160kDa (two ways arrow). Blocking peptide subtracted line scan (BP sub line scan) was calculated by measuring pixel intensity along a line drawn down the lanes, normalizing to a background at high molecular weight, and subtracting the values measured for the corresponding lanes with blocking peptide (blue for TRPM8-GFP lane; orange for GFP). Arrow in the line scan indicates a full-length TRPM8-GFP protein.* ***B****. Western blot of washed platelet lysates from three healthy donors. Line scan was calculated as in* ***A****. Arrows indicate potential TRPM8 protein.* ***C****. Representative images of random TRPM8-positive platelets population and CD45 (-/+) staining from one healthy donor by imaging flow cytometry. 20,000 events were measured for each sample. The scale bar is 7 µm.*

***
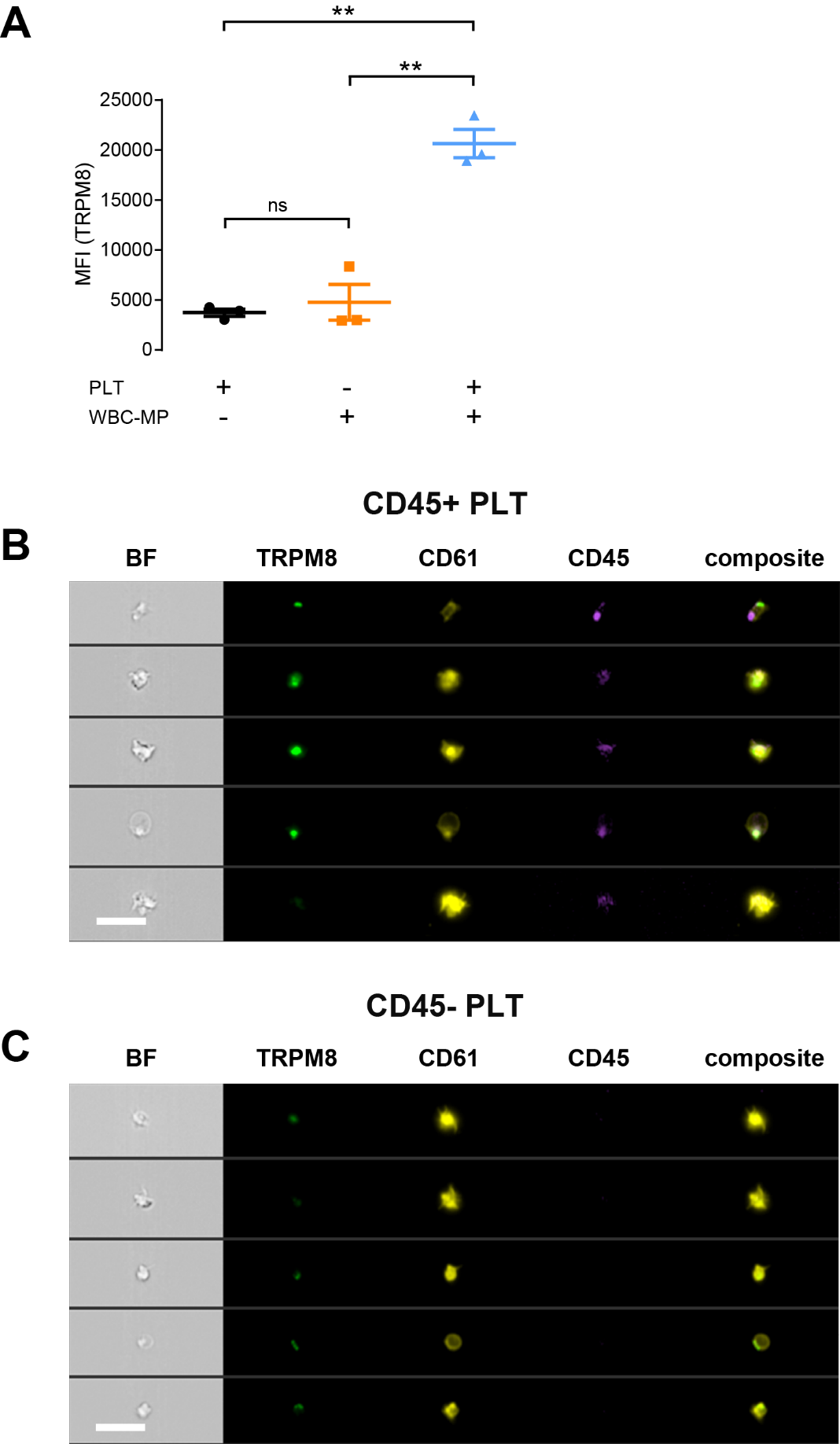
***

***Supplementary Fig 4. (A-C)*** *TRPM8 expression in platelets, WBC-derived microparticles, and platelets incubated with WBC-derived microparticles. Platelet-rich plasma was incubated with WBC-derived microparticles for 15 min at RT. Platelet-rich plasma alone, WBC-derived microparticles alone, and platelet-rich plasma incubated with WBC-derived microparticles were stained with TRPM8 antibody with a secondary FITC-labeled antibody, and the pan WBC, anti-CD45 antibody, along with CD61 and read by conventional flow cytometry* ***(A)*** *and imaging flow cytometry* ***(B-C)****.* ***B.*** *Representative CD45 positive platelets (CD61+ and SSC gated).* ***C.*** *Representative CD45 negative platelets (CD61+ and SSC gated). Data in (A) are shown as individual data points, mean ± standard error of the mean. N=3 independent experiments. **p=0.0029 for PLT versus PLT+WBC-MP, and **p=0.0037 for WBC-MP versus PLT+WBC-MP. Ns= not significant (p=0.88). Statistical analysis was performed using One Way ANOVA with Tukey correction for multiple comparisons and assumed equal sphericity. White bar indicates 7µm.*


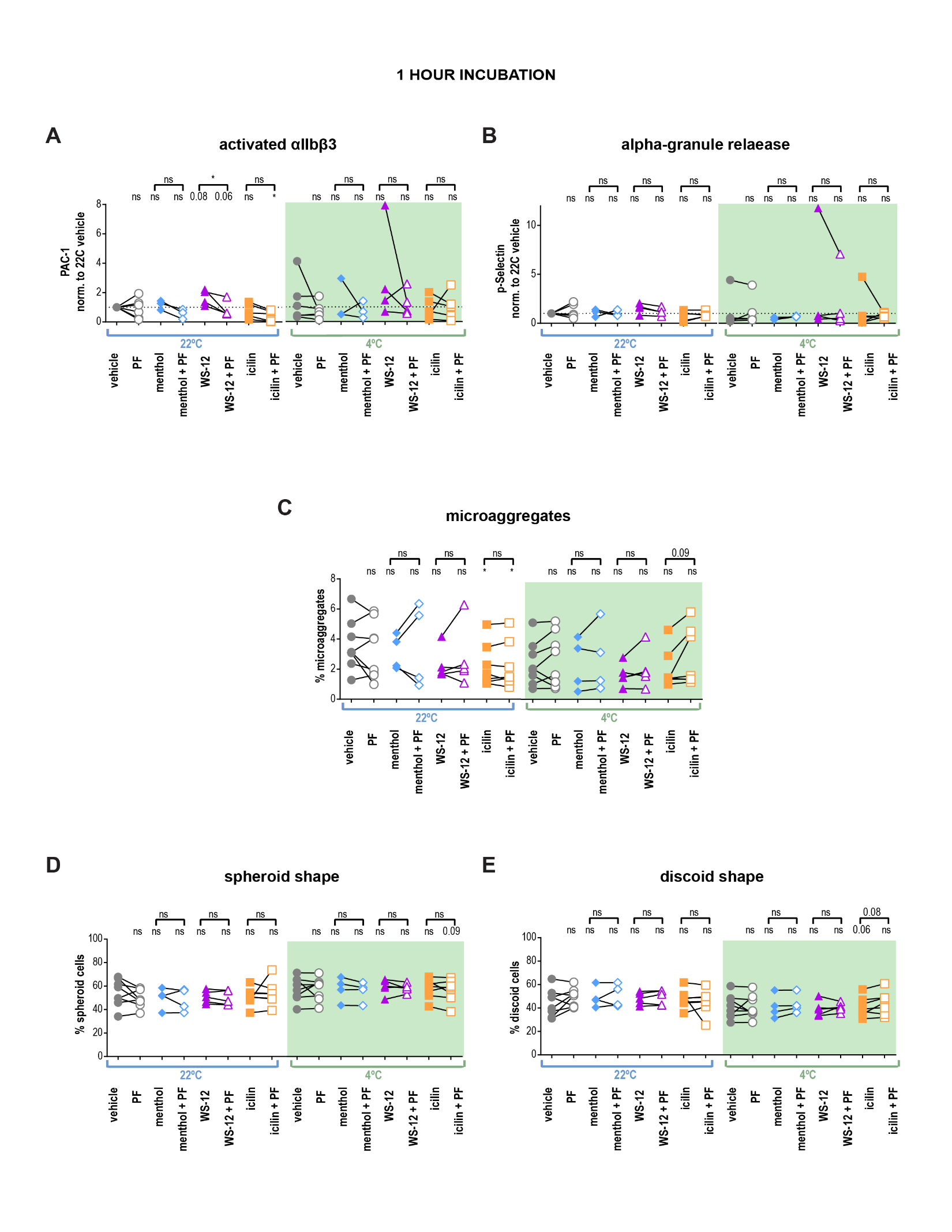


***Supplementary Fig 5. TRPM8 agonists do not lead to activation of the human washed platelets after 1 hour of incubation. (A-B)*** *Human washed platelets were evaluated via flow cytometry.* ***A****.* Integrin αIIbβ3 activation in s*amples stained with PAC-1 fluorescent antibody (MFI normalized to vehicle).* **B.** Alpha granule release as seen from P-selectin externalization *(anti-P-selectin fluorescent antibody MFI normalized to vehicle). Initially, samples were pre-incubated with either vehicle DMSO or PF 05105679 (2 µM) for 5 minutes. Next, samples were treated with either vehicle (Ethanol), menthol (500 µM), WS-12 (2 µM) or icilin (100 µM) for 1 hour at either 22˚C (white background) or 4˚C temperature (green background). Values were normalized to those measured in platelets treated with the vehicle at 22˚C. (****C-E****) Samples were evaluated via imaging flow cytometry.* ***C****. Percent microaggregates in samples treated the same as in A and B. (****D, E****) Percent spheroid (****D****) or discoid (****E****) cells in samples treated same as in A and B. Lines connecting data points indicate the same donor. Statistical analysis was performed using paired Student t-test, where asterisks indicated a p-value lower than 0.05 for *, and “ns” indicates s p-value >0.05. Symbols above brackets indicate paired comparison between treatment groups, and without bars indicate comparison to vehicle.*


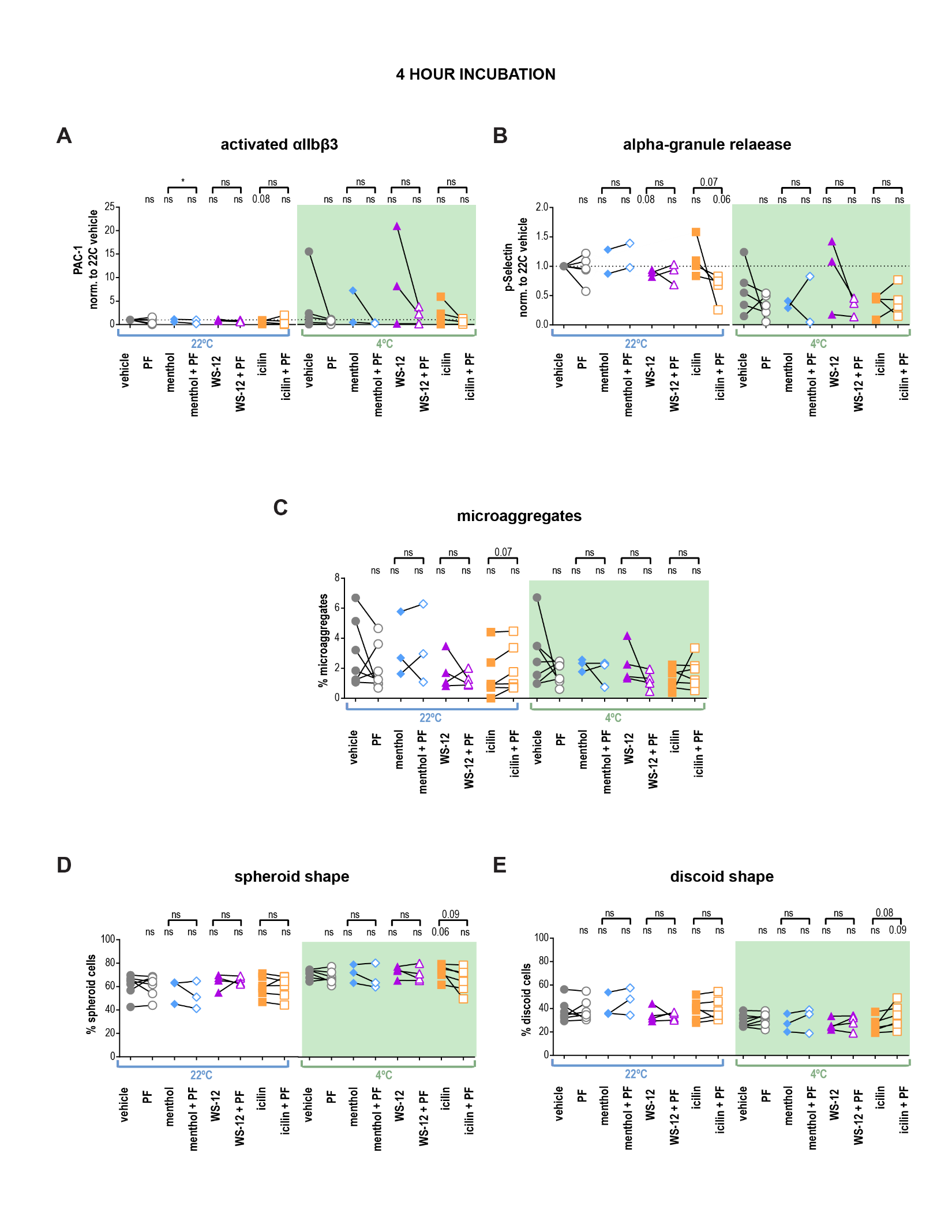


***Supplementary Fig 6. TRPM8 agonists do not lead to activation of the human washed platelets after 4 hours of incubation. (A-B)*** *Human washed platelets were evaluated via flow cytometry.* ***A****. Integrin αIIbβ3* activation in s*amples stained with PAC-1 fluorescent antibody (MFI normalized to vehicle).* **B.** Alpha granule release as seen from P-selectin externalization *(anti-P-selectin fluorescent antibody MFI normalized to vehicle). Initially, samples were pre-incubated with either vehicle DMSO or PF 05105679 (2 µM) for 5 minutes. Next, samples were treated with either vehicle (Ethanol), menthol (500 µM), WS-12 (2 µM) or icilin (100 µM) for 4 hours at either 22˚C (white background) or 4˚C temperature (green background). Values were normalized to those measured in platelets treated with vehicle. (****C-E****) Samples were evaluated via imaging flow cytometry.* ***C.*** *Percent microaggregates in samples treated the same as in A and B. (****D, E****) Percent spheroid (****D****) or discoid (****E****) cells in samples treated the same as in A and B. Lines connecting data points indicate the same donor. Statistical analysis was performed using paired Student t-test, where asterisks indicated a p-value lower than 0.05 for *, and “ns” indicates s p-value >0.05. Symbols above brackets indicate paired comparison between treatment groups, and without bars indicate comparison to vehicle.*


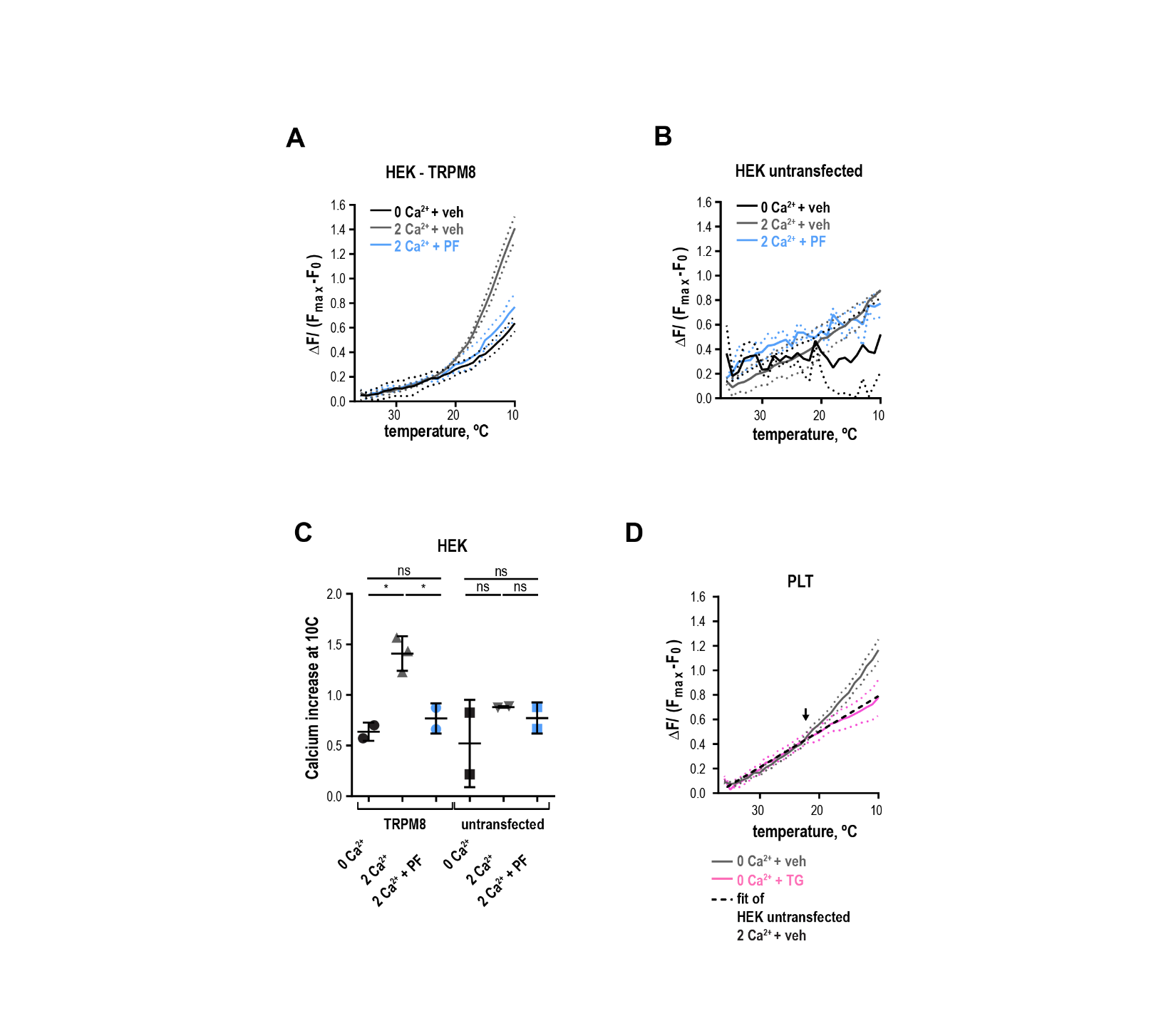


***Supplementary Fig 7. (A-B)*** *Change in Calcium Green™-1 fluorescence levels baseline subtracted and normalized to maximum obtained after addition of calcium ionophore 7 µM A23187. HEK293T/17 cells transfected with TRPM8 (****A****) or empty vector (****B****), (un-transfected) were suspended in HEPES buffered saline containing either 0 mM Ca^2+^ and 100 µM EGTA (black) with vehicle DMSO, 2 mM Ca^2+^ with vehicle DMSO (gray) or 2 mM Ca^2+^ with 2 µM PF 05105679 (blue).* ***C.*** *Quantification of maximal calcium increase at 10˚C in HEK293T/17 cells, n=2.* ***D.*** *The overlay of the linear fit (dashed line, R^2^=0.82) of the average negative control - the un-transfected HEK cells in 2 mM Ca^2+^ (same as in B, gray) and the average calcium response in washed platelets in 0 mM Ca^2+^ and 100 µM EGTA -containing Tyrode’s buffer with vehicle DMSO (gray, same as Fig 7 C) or 5 µM thapsigargin (pink, same as Fig 8 C). Arrow indicates an apparent threshold for platelet activation at ~ 23˚C.*

1. Thapa D, Valente J de S, Barrett B, Smith MJ, Argunhan F, Lee SY, et al. Dysfunctional TRPM8 signalling in the vascular response to environmental cold in ageing. Elife. 2021;10: e70153. doi:10.7554/elife.70153

2. Johnson CD, Melanaphy D, Purse A, Stokesberry SA, Dickson P, Zholos AV. Transient receptor potential melastatin 8 channel involvement in the regulation of vascular tone. Am J Physiol-heart C. 2009;296: H1868–H1877. doi:10.1152/ajpheart.01112.2008

3. Gerhard DS, Wagner L, Feingold EA, Shenmen CM, Grouse LH, Schuler G, et al. The Status, Quality, and Expansion of the NIH Full-Length cDNA Project: The Mammalian Gene Collection (MGC). Genome Res. 2004;14: 2121–2127. doi:10.1101/gr.2596504

4. Bidaux G, Gordienko D, Shapovalov G, Farfariello V, Borowiec A, Iamshanova O, et al. 4TM-TRPM8 channels are new gatekeepers of the ER-mitochondria Ca2+ transfer. Biochimica Et Biophysica Acta Bba - Mol Cell Res. 2018;1865: 981–994. doi:10.1016/j.bbamcr.2018.04.007

5. Bidaux G, Borowiec A, Gordienko D, Beck B, Shapovalov GG, Lemonnier L, et al. Epidermal TRPM8 channel isoform controls the balance between keratinocyte proliferation and differentiation in a cold-dependent manner. Proc National Acad Sci. 2015;112: E3345–E3354. doi:10.1073/pnas.1423357112

6. Lacroix R, Plawinski L, Robert S, Doeuvre L, Sabatier F, Lizarrondo SM de, et al. Leukocyte- and endothelial-derived microparticles: a circulating source for fibrinolysis. Haematologica. 2012;97: 1864–1872. doi:10.3324/haematol.2012.066167
